# Dorsal and volar approach to managing a giant bilobed bicompartmental lipoma of the hand: case report

**DOI:** 10.1080/23320885.2025.2593035

**Published:** 2025-11-29

**Authors:** Antonioenrico Gentile, Ludovica de Gregorio, Fabrizio Schonauer

**Affiliations:** Plastic Surgery Unit, University of Naples “Federico II”, School of Medicine and Surgery, Naples, Italy

**Keywords:** Hand tumors, lipoma, bicompartimental lipoma, swelling tumors, giant lipoma of the hand

## Abstract

**Background:**

Lipomas are common benign tumors, but giant bicompartmental bilobed lipomas in the hand are rare and pose unique diagnostic and surgical challenges due to the hand’s complex anatomy and the proximity of neurovascular structures.

**Case presentation:**

We report the case of a 67-year-old woman with a slowly enlarging, painless mass in her left hand, located between the second and third metacarpals, with both dorsal and volar extensions. Physical examination revealed a firm, well-defined lesion measuring approximately 5 cm in length, associated with mild paresthesia and decreased range of motion. Magnetic Resonance Imaging (MRI) confirmed a well-encapsulated, hyperintense mass consistent with a benign lipoma, exhibiting bicompartmental extension without signs of malignancy.

A dual approach was employed for complete excision: an S-shaped dorsal incision followed by a volar zigzag incision. Intraoperatively, the lesion demonstrated a bilobed hourglass shape crossing through a constriction ring formed by surrounding anatomical structures. Meticulous dissection enabled safe en bloc removal while preserving the extensor tendons and common digital nerves.

The postoperative course was uneventful. The patient resumed active motion two weeks postoperatively, with full recovery of hand function and no recurrence at 6-month follow-up. Histopathological examination confirmed a spindle cell lipoma with no malignant features.

**Conclusion:**

This case highlights the importance of preoperative imaging, surgical planning, and a dual dorsal-volar approach for managing complex lipomas of the hand. Tailoring the surgical strategy to the lesion’s anatomy allows complete excision while minimizing the risk to vital structures and optimizing both functional and cosmetic outcomes.

## Introduction

Hands represent only 2% of the total body surface area and 1.2% of the total body weight, accounting for 15% of all soft tissue tumors. 95% of hand tumors without skin involvement are benign, whereas malignant soft tissue tumors of the hand are rare and represent only 2% of all hand lesions [1].

Lipomas are the most common benign soft tissue tumors but are infrequent in the hand, accounting for approximately 8% of benign hand tumors [2]. These lesions are most frequently found in the thenar and hypothenar regions [3]. They are typically composed of mature adipocytes and present as slow-growing, painless, and mobile masses. While generally benign, lipomas exceeding 5 cm, defined as “giant lipomas,” [4] warrant special attention due to their potential compression of nerve structures and rare risk of malignant transformation [5]. Advanced imaging modalities such as computed tomography (CT) and magnetic resonance imaging (MRI) are essential for accurately delineating tumor margins, guiding surgical planning, and assessing relationships with critical neurovascular and tendinous structures [6,7]. In the hand, where anatomical density is high, these tools are invaluable for differentiating lipomas from other capsulated lesions and their removal presents significant challenges which necessitates extreme surgical care to avoid functional deficits or cosmetic complications [8].

Bicompartmental bilobed lipomas are rare, particularly when situated close to the metacarpal bones, where critical neurovascular structures and tendons are densely packed. As reported by other authors, lipomas infiltrating the deep layers of the hand are surgically approached and removed from the palmar aspect [4]. In this report, we discuss a case of a giant bicompartmental bilobed lipoma that was removed using a dual approach, dorsal and palmar, highlighting the surgical challenge and emphasizing the critical role of preoperative planning and precise dissection in achieving optimal results.

## Case presentation

A 67-year-old Caucasian woman presented to our Plastic Surgery Unit with a progressively enlarging mass in her left hand with a history of several years. The lesion was located between the second and third metacarpal bones with dorsal and volar extensions. On physical examination, the mass was soft and well-defined, but firm, measuring approximately 5 cm in length and 3 cm in width. The patient reported no pain but complained of paresthesia of the ulnar middle finger and radial middle finger and hand swelling. Hand-specific examination revealed a reduction in range of motion (ROM) at the metacarpophalangeal and interphalangeal joints, with limited finger flexion, extension, and impaired composite grip and lateral pinch strength. Fine motor dexterity was also reduced on functional testing.

Sensory assessment was performed in the cutaneous territories overlying and adjacent to the tumor using light touch, pinprick, and static two-point discrimination tests, demonstrating hypoesthesia in the ulnar and radial aspects of the middle finger.

The Poch sign was not assessed.

Her medical history was unremarkable, and no chronic diseases were identified.

### Investigation

Ultrasound examination of the left hand demonstrated a clinically evident nodular formation located between the second and third digits, measuring approximately 4 cm in diameter. The lesion was avascular and of non-specific echogenic pattern, making a definitive characterization not possible. A clinical and surgical evaluation by the Hand Surgery Unit was therefore recommended for excision and subsequent histopathological analysis. Following this ultrasound assessment, magnetic resonance imaging (MRI) was performed for further characterization and preoperative planning.

**Figure 1.**
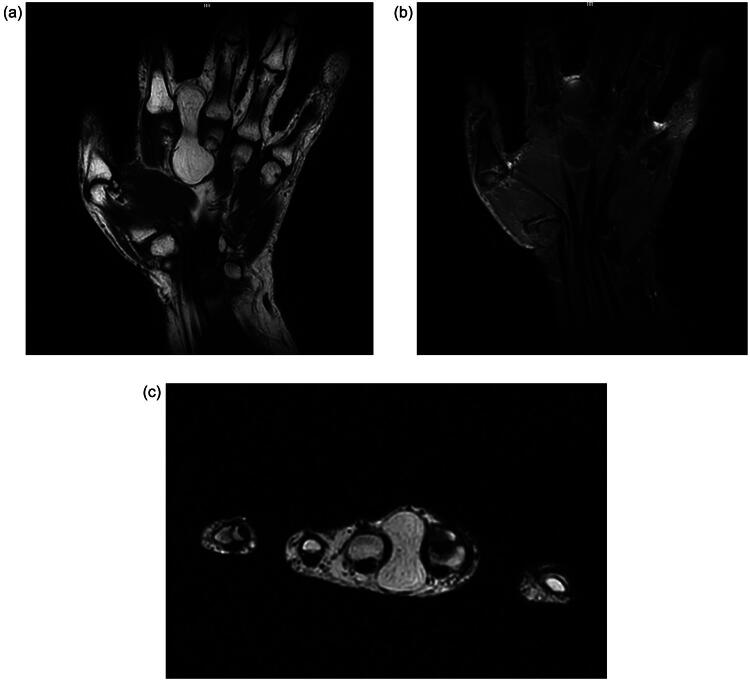
Magnetic Resonance Imaging (MRI) of the right hand demonstrating a well-defined, encapsulated soft tissue mass with homogeneous high signal intensity, consistent with a lipoma. (a) Coronal T1-weighted image shows a hyperintense, bilobed lesion located between the second and third metacarpophalangeal joints, consistent with adipose tissue. (b) Coronal STIR image confirms persistent high signal intensity without fat suppression, supporting the lipomatous nature of the lesion. (c) Axial T1-weighted image reveals the multicompartmental extension of the mass, displacing surrounding structures but without evidence of invasion.

MRI of the left hand revealed a well-defined, encapsulated soft tissue mass located in the intermetacarpal space between the second and third digits at the level of the metacarpophalangeal joints (Figure 1(a–c)). The lesion measured approximately 5 cm in length and 3.5 cm in width.

The mass demonstrated multicompartmental extension, occupying adjacent anatomical compartments while maintaining well-defined margins. On T1-weighted sequences, it exhibited a homogeneously high signal intensity, consistent with fatty tissue. The signal remained hyperintense on T2-weighted and STIR sequences with no evidence of signal suppression, supporting the composition of mature adipose tissue. There were no internal septations, nodular components, or areas of enhancement that would raise suspicion of atypical lipomatous tumors or liposarcomas. It appeared to displace, but not infiltrate, the surrounding soft tissues and neurovascular structures. No bone erosion or periosteal reaction was observed. The adjacent tendons and flexor sheath compartments were intact and no associated inflammatory changes were observed. The overall imaging features were consistent with those of a benign multicompartmental lipoma.

### Surgical management

Written informed consent was obtained from the patient for participation in the study and for the publication of all anonymized data and accompanying images.

Surgery was performed under median and ulnar nerve regional blocks with tourniquet control.

Although the WALANT (Wide Awake Local Anesthesia No Tourniquet) technique was considered, it was ultimately not utilized for this case. A regional nerve block rather than local infiltration was selected to provide prolonged postoperative analgesia. Additionally, the use of a tourniquet was preferred to guarantee an avascular surgical field, providing optimal visualization of critical structures, including neurovascular bundles, given the close relationship of the lipoma to these tissues. The patient remained awake throughout the procedure, allowing real-time monitoring of hand function and tendon integrity during tumor dissection.

**Figure 2.**
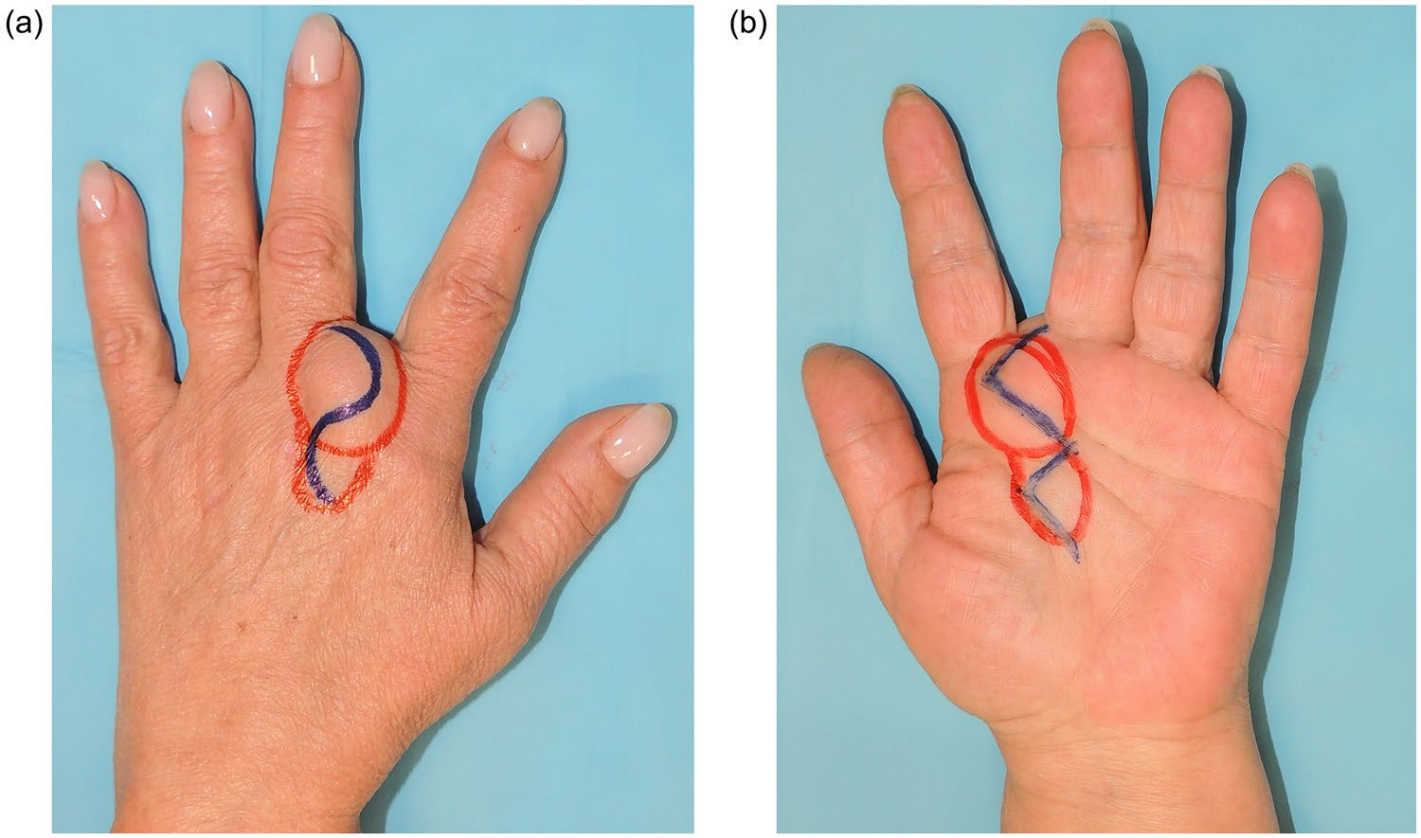
**a.** Pre-operative marking of the S shaped incision on the dorsal side of the giant lipoma. **b.** Pre-operative marking of the zigzag incision on the volar side.

**Figure 3.**
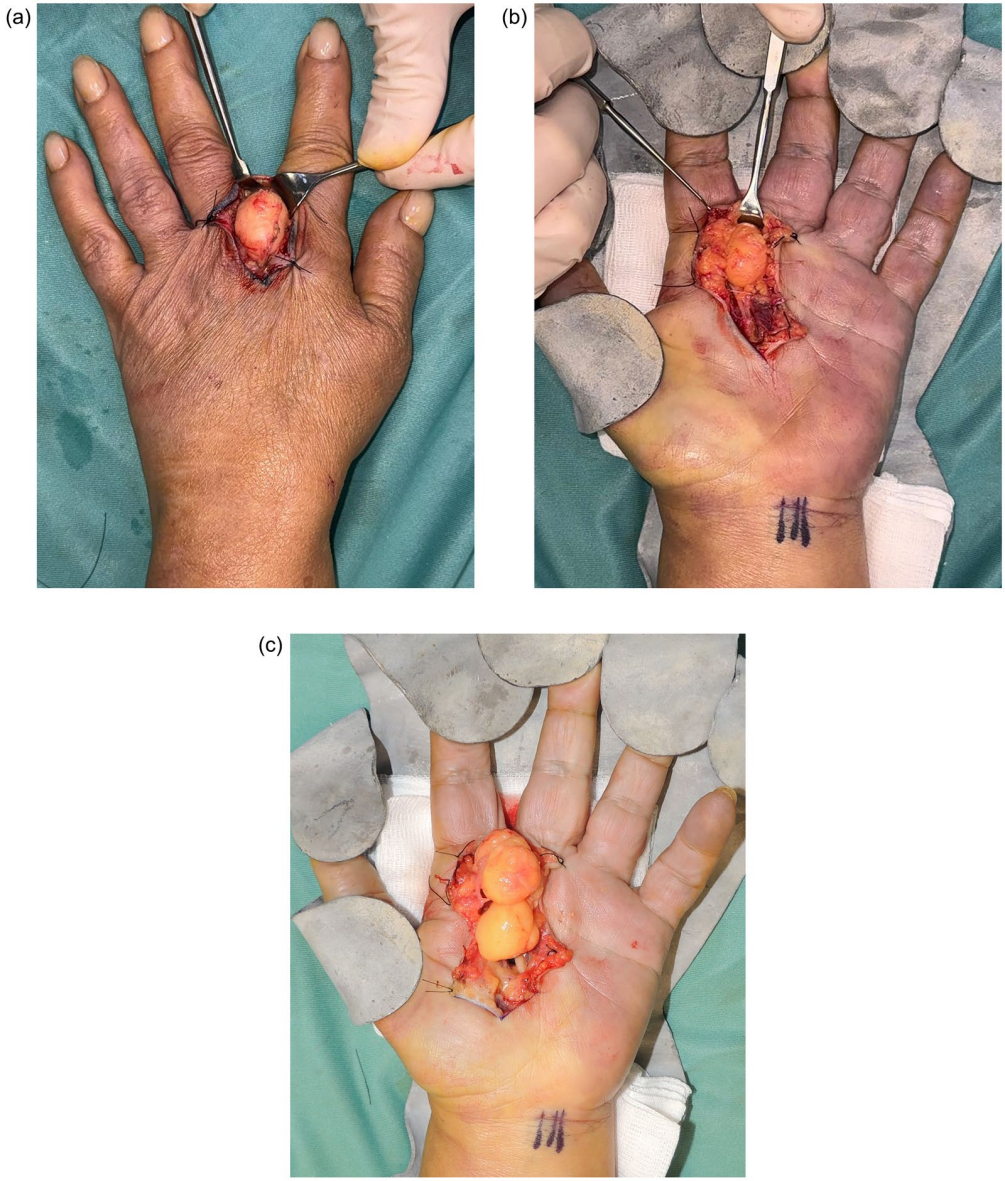
**a.** Intraoperative view of the dorsal approach with optimal exposure for dissection of the lesion, ensuring the preservation of extensor tendons and dorsal neurovascular structures. **b**. Intraoperative volar approach: the lesion is seen passing through the constriction ring formed by the bifurcation of the second and third common digital nerves. **c**. Intraoperative view of the volar approach. The bilobed nature of the lesion and its volar extension is evident, once freed by the second and third common digital nerves’ bifurcation ring.

**Figure 4.**
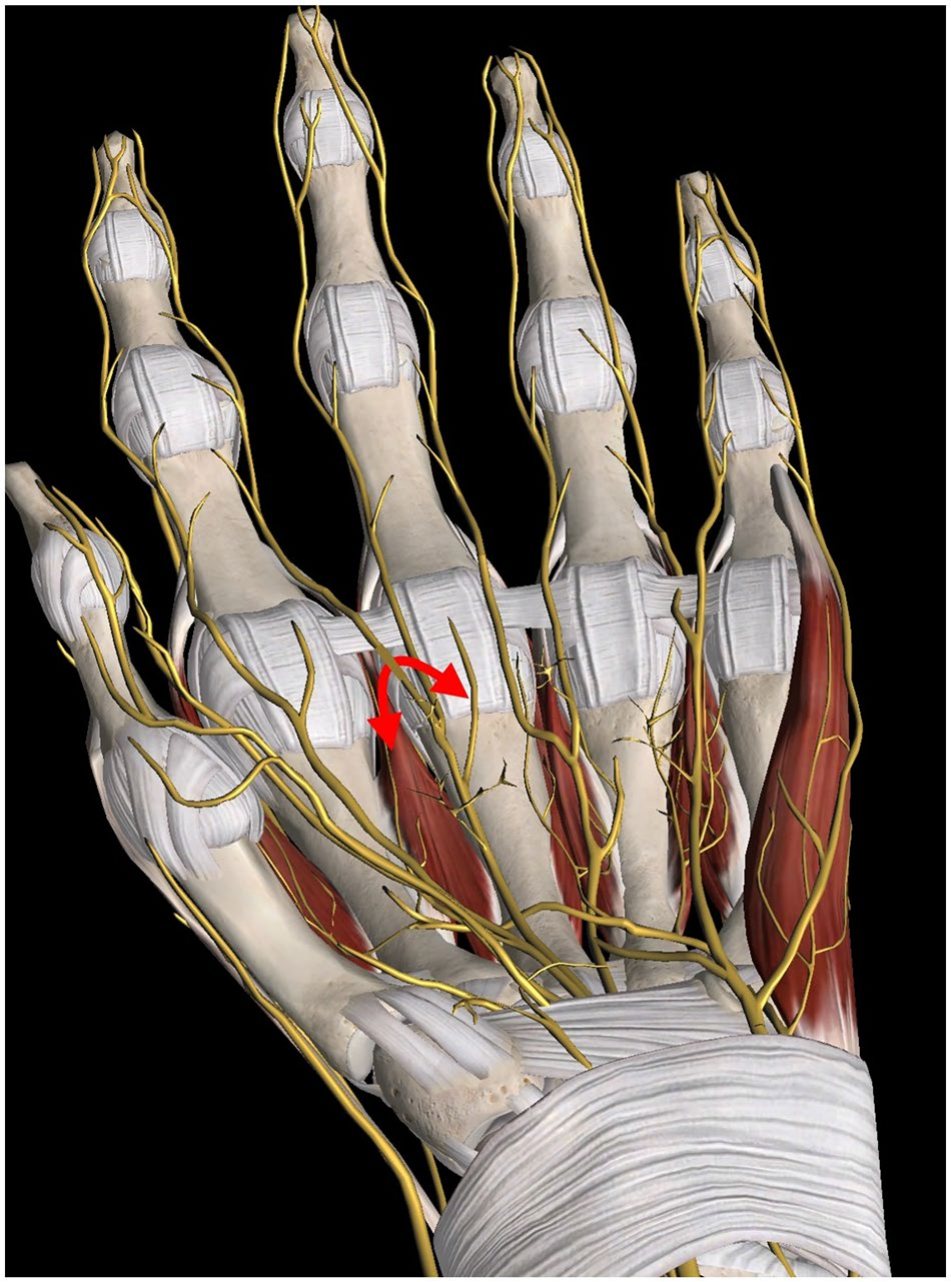
Anatomical representation of the constriction ring through which the lesion extended in the volar and the dorsal aspect of the left hand. The red arrow indicates the pathway of the lipomatous mass passing through the ring, which is defined dorsally by the second deep transverse metacarpal ligament, volarly by the second web space common palmar digital nerve, ulnarly by the metacarpophalangeal joint of the third digit, and radially by the metacarpophalangeal joint of the second digit, explaining the bilobed, bicompartmental morphology of the lesion.

**Figure 5.**
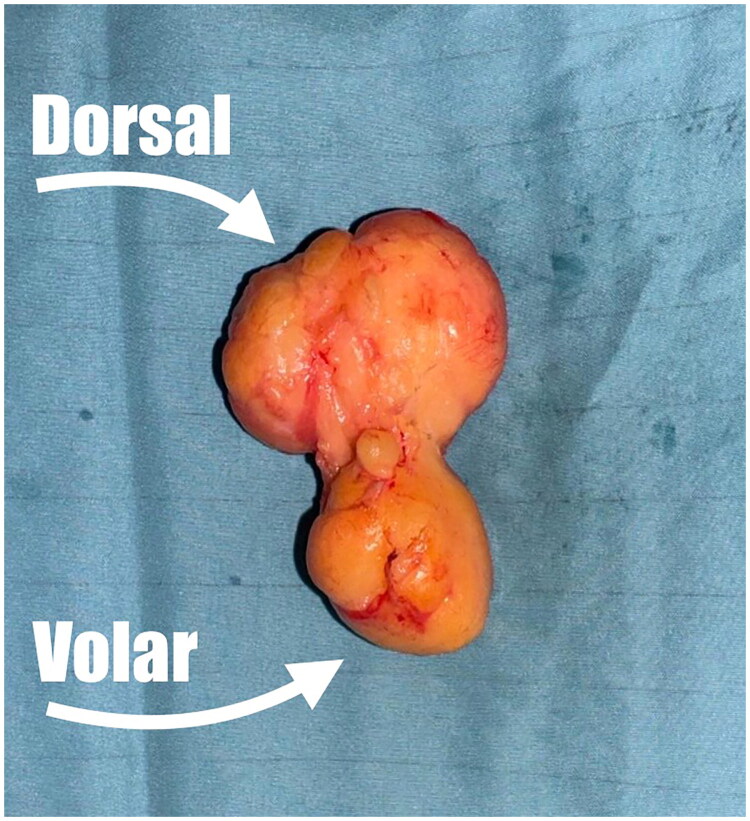
Macroscopic view of the excised lipoma, measuring 5 cm at its largest dimension, clearly showing its bilobed and bicompartmental morphology. The dorsal and volar components are indicated by arrows, corresponding to the respective compartments of the *hand.*

The anatomical complexity and multicompartmental position of the lesion required a dual surgical approach. Preoperative markings of both the dorsal and volar aspects were performed to plan the incisions (Figures 2a and 2b). The dorsal compartment was first accessed *via* an S-shaped incision over the second intermetacarpal space, which provided optimal exposure for dissection (Figure 3a). The lesion was carefully dissected between the index and middle fingers, while preserving the extensor tendons and dorsal neurovascular structures. The hand was turned after a lead hand splint was positioned; approach to the volar compartment was performed through a zigzag incision over the second inter-metacarpal space, allowing access to the palmar portion of the mass (Figure 3b). Upon dissection, the bilobed nature of the tumor was confirmed (Figure 3c), with its volar extension traversing the interdigital space and **lying in close relationship to** the common digital nerves of the second intermetacarpal space, and its branching due to the digital nerve. The dual approach facilitated en bloc resection of the lesion, which presented an hourglass-like appearance. This morphology was attributed to the lesion crossing from the volar to dorsal aspect through a constriction ring. This ring was defined dorsally by the second deep transverse metacarpal ligament, volarly by the second web space common palmar digital nerve, ulnarly by the metacarpophalangeal joint of the III digit, and radially by the metacarpophalangeal joint of the II digit (Figure 4). The lesion measured 5 and 3 cm in length and width, respectively (Figure 5). The neurovascular structures were preserved throughout the procedure. After tourniquet release, careful bipolar hemostasis was achieved, a Penrose drain was placed to manage postoperative fluid accumulation, and a layered skin closure was performed. Immediately after surgery, a soft bandage was applied and the arm was kept elevated for 10 days using a sling.

### Ethical approval

This study was conducted in accordance with the Declaration of Helsinki. Ethical approval was not required for this case report in accordance with the policies of the institutional ethics committee of the University of Naples “Federico II”, as it involves a retrospective description of a single clinical case without experimental intervention.

### Post-operative course and histopathology

**Figure 6.**
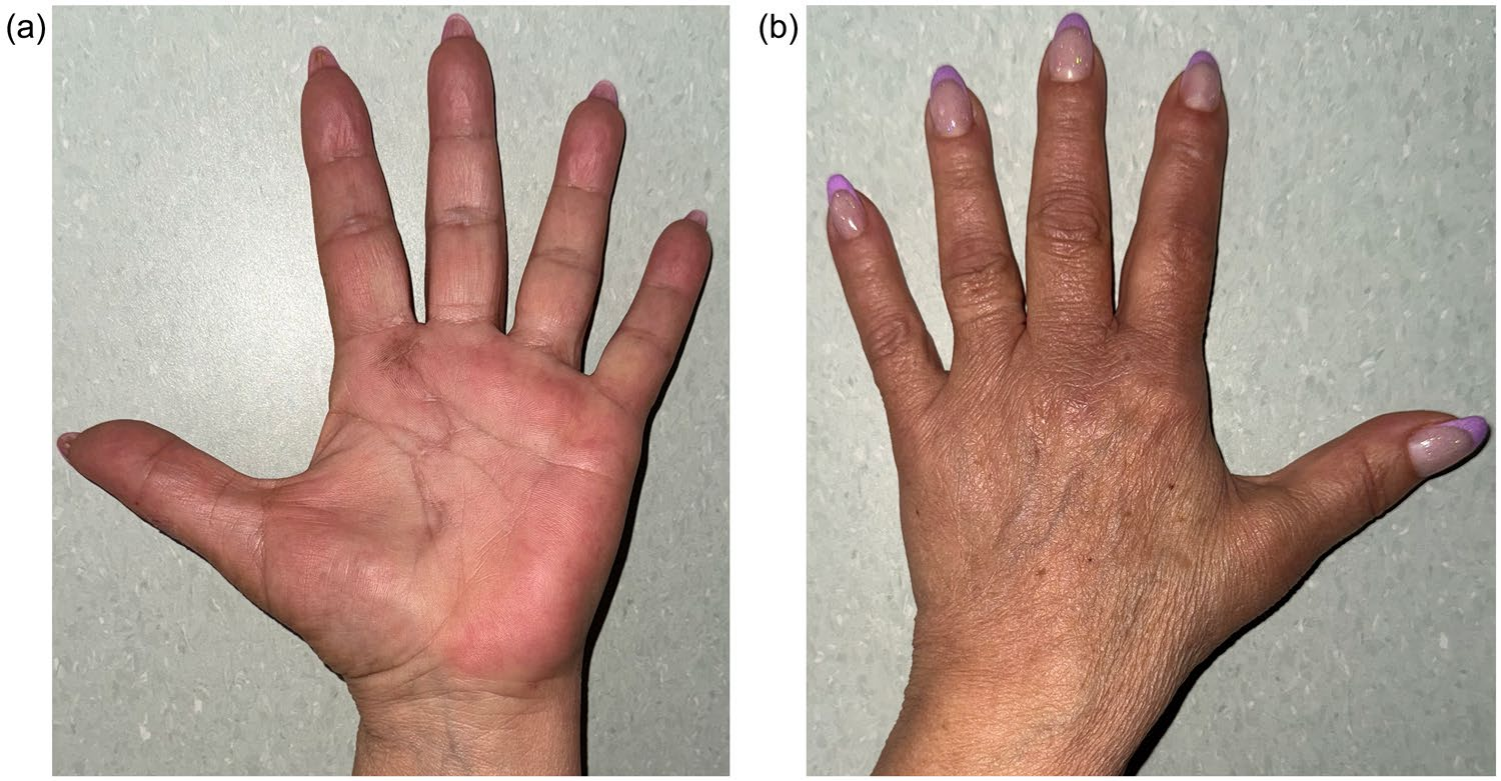
**a.** Post-operative dorsal aspect of the hand at 6 months, showing a well-healed S-shaped scar without visible signs of recurrence. **b.** Post-operative volar aspect of the right hand at 6 months, showing a well-healed zigzag scar without visible signs of recurrence.

No post-operative complications were experienced by any of the patients. Fifteen days after the surgery, the sutures were removed, and the patient started an early active mobilization protocol, which was continued for 6 weeks. After 6 months of follow-up, there was no sign of recurrence, and the patient recovered well with full ROM. of flexors and extensors. The patient experienced an uneventful recovery, retained full hand function, and demonstrated excellent cosmetic outcomes (Figure 6a and b).

Histopathology revealed mature, capsulated adipose tissue with focal presence of fibrous septa and focal areas with spindle cell components (CD34+, p53-), compatible with a lipoma with spindle cell lipoma.

## Discussion

Hand swelling can represent a wide spectrum of differential diagnoses, especially when evaluating well-circumscribed and encapsulated lesions. The most frequent benign entities include ganglion cysts, epidermoid inclusion cysts, fibromas of the tendon sheath, and schwannomas, which may mimic lipomatous tumors both clinically and radiologically [1,9]. In some cases, giant cell tumors of tendon sheath or low-grade sarcomas such as atypical lipomatous tumors and liposarcomas must also be considered in the differential, underlining the importance of MRI in distinguishing these lesions [6].

Although malignant transformation of hand lipomas is exceedingly rare, the possibility of atypical lipomatous tumor or well-differentiated liposarcoma must be considered in large or rapidly growing lesions. Boanimbek et al. [5] reported a rare case of primary liposarcoma involving the fibular head, illustrating that liposarcomas can present as deceptively benign masses. Similarly, Changazi et al. [7] described a lipoblastoma mimicking a benign swelling. These reports highlight the importance of histopathological confirmation after excision, as imaging alone cannot completely exclude malignancy [5,7].

Chatterton et al. [10], Singha et al. [11], and Pagonis et al. [12] described a palmar approach for the excision of giant lipomas of the hand, highlighting the risks posed by close relationships with neurovascular structures. Conversely, Bocchiotti et al. [4] advocated a dorsal approach, emphasizing its relative safety and faster postoperative recovery. They suggested a combined approach only when intraoperative findings revealed fragmentation or firm adhesion to the volar aspect. However, the lesion presented by Bocchiotti et al. was located at two-thirds of the total extension within the dorsal compartment, allowing for a single approach. In contrast, our case demonstrates the value of a dual dorsal–palmar approach planned preoperatively. This strategy allowed safe and complete removal of a bilobed bicompartmental lesion while preserving both tendinous and neurovascular structures. Unlike previous reports, in which the surgical approach was often chosen intraoperatively based on the lesion’s apparent extension, our case highlights the importance of preoperative MRI in defining the surgical plan. This evolution from intraoperative adaptation to image-guided preoperative planning underlines a methodological advance in the management of giant hand lipomas. To our knowledge, this represents the first report in the literature describing a dual approach as the primary surgical plan for such a pathology.

The principles applied in this case - comprehensive imaging, meticulous preoperative planning, precise dissection, and early mobilization - are essential for optimal outcomes in rare hand tumors. These include not only lipomas but also lipoblastomas and liposarcomas [7,13]. Early mobilization is critical to prevent joint stiffness and promote functional recovery [14]. Similarly, in other rare tumors such as histiocytoid hemangiomas, thorough preoperative planning and careful dissection have been shown to be key determinants of surgical success [15]. Ultimately, this case adds to the limited body of literature on giant lipomas of the hand by providing evidence that meticulous imaging, thoughtful planning, and a tailored dual approach can ensure both oncological safety and functional preservation, principles that remain central in modern hand surgery.

## Conclusion

Giant bicompartmental bilobed lipomas of the hand require meticulous preoperative evaluation and precise surgical planning. Advanced imaging is essential to delineate the tumor’s relationship with critical neurovascular structures. This report is unique in demonstrating that a combined dorsal–volar surgical approach can be effectively applied to the excision of a giant multicompartmental hand lipoma. To our knowledge, such a dual approach has not previously been described for the management of this pathology, and in our case, it allowed complete tumor removal while preserving critical neurovascular structures.
